# The proteome of IVF-induced aberrant embryo-maternal crosstalk by implantation stage in ewes

**DOI:** 10.1186/s40104-019-0405-y

**Published:** 2020-01-14

**Authors:** Qianying Yang, Wei Fu, Yue Wang, Kai Miao, Haichao Zhao, Rui Wang, Min Guo, Zhilong Wang, Jianhui Tian, Lei An

**Affiliations:** Key Laboratory of Animal Genetics, Breeding and Reproduction of the Ministry of Agriculture, National Engineering Laboratory for Animal Breeding, College of Animal Science and Technology, China Agricultural University, Beijing, 100193 China

**Keywords:** Conceptus, Embryo-maternal crosstalk, Endometrial C areas, Endometrial IC areas, Ewes, HPLC-MS/MS, IVF, Proteome

## Abstract

**Background:**

Implantation failure limits the success of *in vitro* fertilization and embryo transfer (IVF-ET). Well-organized embryo-maternal crosstalk is essential for successful implantation. Previous studies mainly focused on the aberrant development of *in vitro* fertilized (IVF) embryos. In contrast, the mechanism of IVF-induced aberrant embryo-maternal crosstalk is not well defined.

**Results:**

In the present study, using ewes as the model, we profiled the proteome that features aberrant IVF embryo-maternal crosstalk following IVF-ET. By comparing *in vivo* (IVO) and IVF conceptuses, as well as matched endometrial caruncular (C) and intercaruncular (IC) areas, we filtered out 207, 295, and 403 differentially expressed proteins (DEPs) in each comparison. Proteome functional analysis showed that the IVF conceptuses were characterized by the increased abundance of energy metabolism and proliferation-related proteins, and the decreased abundance of methyl metabolism-related proteins. In addition, IVF endometrial C areas showed the decreased abundance of endometrial remodeling and redox homeostasis-related proteins; while IC areas displayed the aberrant abundance of protein homeostasis and extracellular matrix (ECM) interaction-related proteins. Based on these observations, we propose a model depicting the disrupted embryo-maternal crosstalk following IVF-ET: Aberrant energy metabolism and redox homeostasis of IVF embryos, might lead to an aberrant endometrial response to conceptus-derived pregnancy signals, thus impairing maternal receptivity. In turn, the suboptimal uterine environment might stimulate a compensation effect of the IVF conceptuses, which was revealed as enhanced energy metabolism and over-proliferation.

**Conclusion:**

Systematic proteomic profiling provides insights to understand the mechanisms that underlie the aberrant IVF embryo-maternal crosstalk. This might be helpful to develop practical strategies to prevent implantation failure following IVF-ET.

## Background

In mammals, well-orchestrated embryo-maternal crosstalk during the implantation stage is of prime importance to establish and maintain pregnancy. Despite the diversity of implantation and placentation strategies, the reciprocal interaction that occurs between the embryos and the maternal uterine endometria is shared among species. Briefly, pregnancy recognition signals from peri-implantation embryos act on the endometrium in a paracrine manner to stimulate uterine receptivity, which supports conceptuses development [1–3]. Aberrant crosstalk impairs embryo development and endometrium receptivity, thus leading to implantation failure, which is the most prominent factor for pregnancy loss following both natural conception and when using assistant reproductive technology (ART) [4, 5].

Until now, the success rate following *in vitro* fertilization and embryo transfer (IVF-ET) has remained disappointingly low among species [6, 7]. Implantation failure remains the biggest barrier that limits the success rate. Aiming to improve the IVF success rate, numerous studies have been performed that focused on the mechanisms responsible for impaired development potential of IVF embryos, including many genetic and cellular changes, such as epigenetic modifications [8, 9], genetic information processing [10], energy metabolism [11], and cytoskeleton organization [12]. Based on these observations, strategies have been applied to correct these aberrations, thus enhancing IVF embryo developmental potential [9, 12–15].

However, endometrial receptivity, an early sensor of embryo implantation signals and a prerequisite of successful pregnancy [16], has not been fully considered as a target for improving IVF outcomes. In fact, limited prior work demonstrated that the endometrium responds aberrantly to *in vitro* fertilized or cloned embryos, compared with its response to *in vivo* fertilized embryos [1, 4, 17]. This suggests that not only impaired embryo quality, but also the aberrant endometrial receptivity induced by IVF embryos, might contribute to implantation failure following IVF-ET. In clinical practice, treatment of IVF/ET patients with N-acetyl-*L*-cysteine (NAC), melatonin and selenomethionine during the peri-implantation stage has been used to improve IVF outcomes, suggesting that the maternal endometrium can be used as target, and provides alternative strategies for improving the IVF success rate [18]. However, the mechanism, underlying aberrant embryo-maternal crosstalk following IVF-ET is not well defined.

High-throughput methodologies, such as transcriptomic and proteomic analyses, have been frequently applied to profile the cellular responses of embryos and the endometria by implantation stage in various animal models, including sheep [19–22], cattle [23–25], mice [26, 27], pigs [28], and humans [29, 30]. Among these, ruminants have been used extensively as models to explore embryo-maternal interactions by implantation stage [31, 32]. Distinct from rodents or humans, the embryo-maternal crosstalk in ruminants is characterized by interaction occurring in both the caruncular (C) and intercaruncular (IC) areas. Aglandular C areas serve as the sites of superficial attachment and placentation. Glandular IC areas, which contain large numbers of branched and coiled uterine glands, are mainly responsible for the synthesis and secretion of histotroph [33, 34]. In the present study, using ewes as the model, we profiled the proteome of aberrant embryo-maternal crosstalk following IVF-ET. Compared with the high-throughput analysis focusing on mRNA expression, proteomic analysis provides a more direct and accurate understanding, because proteins are the executors of most biological programs [35]. Therefore, the proteome of the conceptuses produced by IVF and their matched endometria by implantation stage will provide a novel and detailed reference to understand the mechanisms that underlie aberrant IVF embryo-maternal crosstalk, and will provide important clues to improve IVF outcomes from both the embryonic and maternal sides.

## Methods

### Animals and treatment

The experiments were performed in accordance with the Guide for the Care and Use of Agricultural Animals in Agricultural Research and Teaching, and all procedures were approved by the Institutional Animal Care and Use Committee at China Agricultural University (Beijing, China). Chinese Small Tail Han ewes with normal estrous cycles were selected for the present study. The procedures of estrous synchronization, superovulation, artificial insemination (AI), collection, and transfer of IVO blastocysts were performed as described in our previous study [22].

### IVF-ET processes

The methods for IVF were conducted as described by Ptak et al. [8]. Following collection of sheep ovaries at slaughter, oocytes were aspirated using 12 G needles and placed into oocyte wash buffer TCM199-hepes (Sigma, St. Louis, MO, USA), 1 mg/mL polyvinyl alcohol (Sigma), 10–20 μg/mL heparin sodium (Sigma), P/S (100 IU/mL penicillin (Sigma) and 100 IU/mL streptomycin (Sigma). Oocytes, surrounded by integrated granulosa cells and with evenly granulated cytoplasm were selected for *in vitro* maturation (IVM). Oocytes from donors were incubated in maturation medium TCM199–HCO_3_ (Sigma) containing 10% FBS (fetal bovine serum; GIBCO, Grand Island, NY, USA), 10 μg/mL FSH (follicle stimulating hormone, Vetrepharm, Concord, Canada), 10 μg/mL LH (luteinizing hormone, Sigma), 1 μg/mL estradiol (Sigma), 10 ng/mL epidermal growth factor (EGF), 0.1 mmol/L cysteamine [36, 37] (Sigma), and P/S covered with mineral oil and incubated in a humidified atmosphere of 5% CO_2_ at 38.6 °C for 24–26 h. Matured oocytes were gently denuded of granulosa cells by 0.05% hyaluronidase (Sigma) and transferred into 50 μL drops of synthetic oviductal fluid (SOF, Sigma) enriched with 20% (v/v) serum, 2.9 mmol/L of Ca lactate, and 16 mmol/L of isoproterenol. Ram semen was thawed and capacitated, then *in vitro* fertilization (IVF) was performed in drops using sperm at a final concentration about 2 × 10^6^ spermatozoa/mL and 15–20 oocytes per drop. After 20 h, zygotes were transferred into a four-well plate with 500 μL of *in vitro* culture medium [SOF enriched with bovine serum albumin (BSA)], covered with mineral oil, and incubated a humidified atmosphere of 5% CO_2_, 5% O_2_, and 90% N_2_ at 38.6 °C. Two well-developed day 6.5 IVF blastocysts were transferred to each synchronized recipient ewe, thus, the day of fertilization was defined as day 0. To minimize the differences caused by the surgical procedure and embryo quality, we selected a skillful technician to perform the surgical procedure, and only good-quality blastocysts (Grade 1) were transferred to synchronized recipient ewes.

### Sample collection

We collected good-quality IVO embryos from thirty donors at day 6.5 of pregnancy, and day 6.5 IVF embryos from the IVF process. Then, two well-developed blastocysts were transferred per synchronized recipient ewe (forty-eight synchronized ewes for the IVO group, thirty-eight for the IVF group). Sampling procedures were similar to the methods detailed in our previous study [22]. Briefly, all recipients were slaughtered at day17 of pregnancy, then their uteri were collected and the conceptuses were flushed out with phosphate-buffered saline (PBS). Thirty-seven recipients in the IVO group and twenty recipients in the IVF group had filamentous conceptuses. The endometrial caruncular (C) and intercaruncular (IC) areas were collected and processed as described by Attia et al. [1]. Opening the ipsilateral uterine horn longitudinally by scissors, the C areas were first carefully cut out and collected, and then the IC areas were sampled. The same technician took the samples from the IVO and IVF group, which were stored at liquid nitrogen until further analysis (Additional file 1: Figure S1A).

### Protein extraction

We equally divided thirty-six IVO samples (or eighteen IVF samples) into three pools, with twelve IVO samples in each pool (six IVF samples per pool). Each pool was ground to powder in liquid nitrogen and stored overnight at − 20 °C after adding a five-fold volume of chilled acetone containing 10% trichloroacetic acid (TCA) and 10 mmol/L dithiothreitol (DTT). The samples were then centrifuged at 4 °C, 16,000×*g* for 20 min and the supernatant was discarded. The precipitates were mixed with 1 mL chilled acetone containing 10 mmol/L DTT and centrifuged at 4 °C, 20,000×*g* for 30 min after storing for 30 min at − 20 °C. Centrifugation was repeated several times until the supernatant was colorless. The pellets were air-dried, dissolved in lysis buffer containing 1 mmol/L phenylmethanesulfonyl fluoride (PMSF), 2 mmol/L ethylenediaminetetraacetic acid (EDTA), and 10 mmol/L DTT and sonicated at 200 W for 15 min before being centrifuged at 30,000×*g* at room temperature for 30 min. The protein concentration in the supernatant was then detected by using the Bradford method.

### Peptide digestion

Proteins (50 μg) were taken from each sample, and isopycnic samples were prepared by adding 8 mol/L urea solution. To reduce disulfide bonds, the samples were incubated with 10 mmol/L DTT at 56 °C for 1 h, and then cysteine bonding was blocked using 55 mmol/L iodoacetamide (IAM) in a dark room for 45 min. Thereafter, each sample was diluted 8-fold with 50 mmol/L ammonium bicarbonate and digested with Trypsin Gold at a protein: trypsin ratio of 20:1 at 37 °C for 16 h. Following desalting using a Strata X C18 column (Phenomenex, Torrance, CA, USA), the samples were vacuum dried. Peptides generated from digestion were directly loaded for liquid chromatography/electrospray ionization tandem mass spectroscopy (LC-ESI-MS/MS) analysis.

### LC-ESI-MS/MS analysis with LTQ-orbitrap collision induced dissociation (CID)

Each sample was resuspended in buffer A [2% acetonitrile (ACN), 0.1% formic acid (FA)] and centrifuged at 20,000×*g* for 10 min. The final peptide concentration for each sample was approximately 0.5 μg/mL. The digested samples were fractionated using a Shimadzu LC-20 AD nano-high performance liquid chromatography (HPLC) system (Shimadzu, Kyoto, Japan). Each sample (10 μL) was loaded by the autosampler onto a 2-cm C18 trap column (200 μm inner diameter), and the peptides were eluted onto a resolving 10 cm analytical C18 column (75 μm inner diameter) prepared in-house. The samples were loaded at a flow rate of 15 μL/min for 4 min, and then a 91-min gradient from 2% to 35% buffer B (98% ACN, 0.1%FA) was run at a flow rate of 400 nL/min, followed by a 5-min linear gradient to 80% buffer B that was maintained for 8 min before finally returning to 2% buffer B within 2 min. The peptides were subjected to nanoelectrospray ionization and then detected by MS/MS in an LTQ Orbitrap Velos (Thermo Fisher Scientific, Bremen, Germany) coupled online to a HPLC system. Intact peptides were detected in the Orbitrap analyzer at a resolution of 60,000 *m*/*z*. Peptides were selected for MS/MS using the CID operating mode with a normalized collision energy setting of 35%, and ion fragments were detected in the LTQ. One MS scan followed by ten MS/MS scans was applied for the ten most abundant precursor ions above a threshold ion count of 5000 in the MS survey scan. Dynamic exclusion was used, with the following parameters: Repeat counts = 2; repeat duration = 30 s; and exclusion duration = 120 s. The applied electrospray voltage was 1.5 kV. Automatic gain control (AGC) was used to prevent overfilling of the ion trap; 1 × 10^4^ ions were accumulated in the ion trap to generate CID spectra. For MS scans, the *m/z* scan range was 350 to 2000 Da.

### Proteomic analysis

MaxQuant software (version 1.1.1.36) was used to analyze the mass spectra. *Bos taurus* is the only well-annotated species with a genomic database with a high degree of homology to sheep. Therefore, we generated one reference protein database by integrating the following databases and sequences of cattle proteins and limited publically available sheep proteins, and removed duplicate proteins: GenBank nr (20110403), Uniprot cow proteins (20110503), sheep proteins (http://www.livestockgenomics.csiro.au/sheep/), and cow proteins (http://genomes.arc.georgetown.edu/drupal/bovine/). The MS/MS data were searched against the reference protein database using the search engine embedded in MaxQuant. Up to two missed cleavages were allowed. The first search was set to 20 ppm, and the MS/MS tolerance for CID was set to 0.5 Da. The false discovery rate (FDR) was set to 0.01 for peptide and protein identifications, which was estimated based on the fraction of reverse protein hits [38, 39]. Proteins were considered identified when at least two peptides were identified, at least one of which was uniquely assignable to the corresponding sequence. In the case of identified peptides that were all shared between two proteins, these were combined and reported as one protein group. To control the false match frequency, the contents of the protein table were filtered to eliminate identifications from the reverse database and common contaminants [40, 41]. The minimum peptide length was set to six amino acids. To perform label-free quantification analysis, the MaxQuant software suite containing an algorithm based on the extracted ion currents (XICs) of the peptides was used. Xcalibur 2.1 (Thermo Scientific) was used as quality control program to check the quality of chromatographs. This specific label-free processing method was performed as described by Waanders et al. [42].

### Cell culture

A human endometrial cancer cell line (Ishikawa, ATCC, USA). Ishikawa cells were grown at 37 °C in DMEM (Hyclone, Logan, UT) supplemented with 10% fetal bovine serum (FBS; Hyclone, Logan, UT) and 1% penicillin/streptomycin (Invitrogen) in a humidified 5% CO_2_ incubator. Cells were treated with H_2_O_2_ (50 μmol/L, 200 μmol/L), NAC (10 μmol/L), and melatonin (10^− 7^ mol/L).

### Immunofluorescence

To detect the abundance and distribution of actin filaments, fluorescein isothiocyanate labeled.

Phalloidin (FITC-phalloidin, Sigma) was used. FITC-phalloidin was prepared in accordance with the manufacturer’s instructions. Briefly, FITC-phalloidin was dissolved as a stock solution (0.1 mg/mL) in dimethyl sulfoxide and stored at − 20 °C. The stock solution was later diluted to the working concentration (5 μg/mL) in PBST (0.2% Triton-X100 in PBS) before use. Human endometrial cancer cells (Ishikawa line) were plated on glass coverslips and fixed with 3.7% formaldehyde for 10 min at room temperature, and then permeabilized in PBST for 20 min at room temperature. After washing three times with PBS at 37 °C for 5 min each, the cells were incubated with FITC- phalloidin overnight at 4 °C. After washing three times with PBS at 37 °C for 5 min, cells were counterstained with 4′,6-diamidino-2-phenylindole (DAPI; Vector Laboratories, Burlingame, CA, USA) for 10 min and mounted on glass-bottomed culture dishes (Wuxi Nest Biotechnology Co, Ltd., Jiangsu, China) with Vectashield mounting medium (Vector Laboratories). Fluorescent signals were acquired on an upright microscope (BX51; Olympus, Tokyo, Japan) using an attached digital microscope camera (DP72; Olympus).

### Total protein detection

The total protein concentration of the IVO and IVF conceptuses, C areas, or IC areas was quantified using an enhanced BCA protein assay kit (Beyotime Biotechnology, Jiangsu, China), and normalized by the weight of the tissues, providing the total protein concentration per gram of tissue.

### Determination of ATP content

The ATP levels in the IVF and IVO conceptuses were detected using an Enhanced ATP Assay Kit S0027 (Beyotime Biotechnology) according to the protocol provided by the manufacturer. In brief, 20 mg of tissues were lysed in ATP lysis buffer, centrifuged for 5 min at 4 °C and 12,000×*g*, and the supernatant was collected. A portion of the supernatant was used to detect the ATP concentration, and the other portion of the supernatant was used to detect the total protein concentration. Finally, the total ATP concentration was normalized by the total protein concentration.

### Data analysis

To facilitate data analysis, all proteins were mapped to the Ensembl *Bos taurus* gene ID. The protein quantification values of the IVO/IVF conceptuses, C areas, and IC areas are shown in Additional file 2: Table S1. Student’s *t*-test was used to detect the significance of the differentially expressed proteins (DEPs), and *P* < 0.1 was considered significant, which would avoid removing putative candidates [43, 44]. Data are presented as mean values ± SEM. DAVID version 6.7 (http://david.abcc.ncifcrf.gov/) enables the generation of specific functional annotations of biological processes affected by treatment from the target gene lists produced in high-throughput experiments [45, 46]. We used DAVID to carry the gene-annotation enrichment analysis. Then, QuickGO (https://www.ebi.ac.uk/QuickGO/) was used to identify the DEPs involved in some enriched processes in DAVID’s GO annotation [47]. Moreover, gene symbols corresponding to DEPs were also sent to the Search Tool for the Retrieval of Interacting Genes/Proteins (STRING version 9; http://string.embl.de/ [48]) to build a network using edge information from three separate forms of evidence: Databases, experiments, and text mining. We used 0.4 (medium confidence), the default and recommended option to construct protein-protein interaction networks by the STRING on-line platform, as the value for edge confidence provided by STRING. To assess the similarities of the different replicates, and to obtain a visual understanding of the relationship between the different experimental groups, we used REVIGO (http://revigo.irb.hr/) to summarize long lists of Gene Ontology terms and visualized the remaining terms in interactive graphs [49]. Phenotype annotations of DEPs were analyzed based on the MGI database (Mouse Genome Informatics, http://www.informatics.jax.org/phenotypes.shtml). The CLUSTER 3.0 data analysis tool was used to carry out hierarchical clustering based on the clusters of protein expression profiles of different technical and biological replicates. Unsupervised hierarchical clustering analysis was carried using the “hclust” function in R (R version 3.5.1. https://www.R-project.org/.). The *P*-value of Student’s *t*-test was calculated by GraphPad Prism software or R for individual analysis.

## Results and discussion

### The proteome of conceptuses and endometria from the IVO and IVF groups

**Fig. 1:**
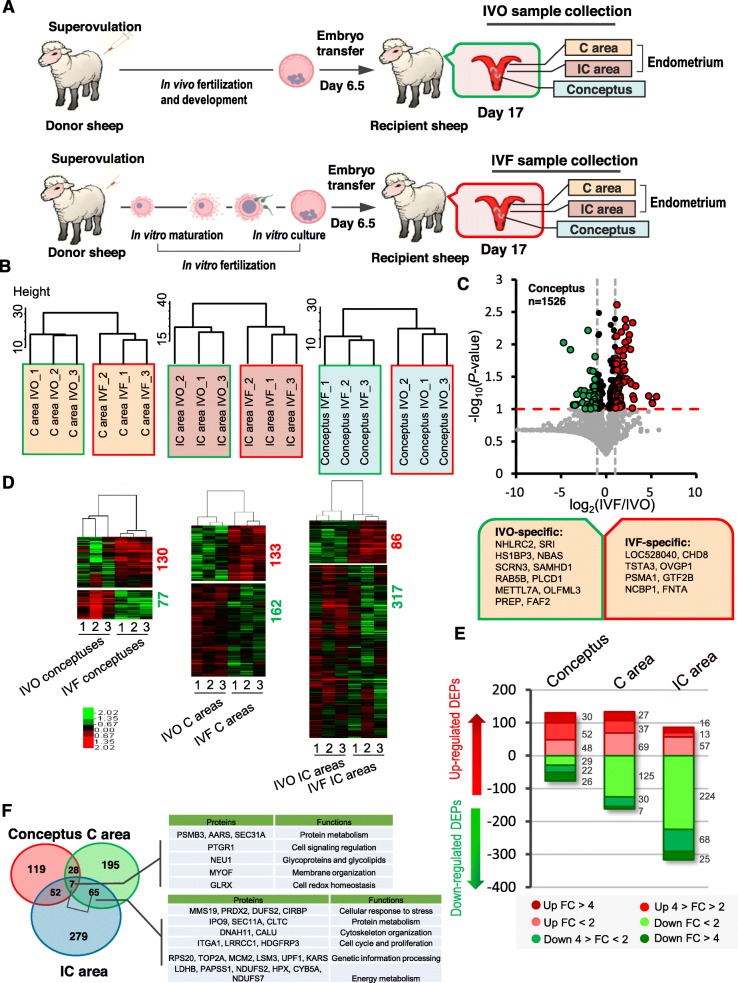
The proteome of conceptuses and endometria from the IVO and IVF Groups. **a** Schematic illustration of the experimental design to study the proteome of IVF embryo-maternal crosstalk. **b** Unsupervised clustering of protein expression patterns in IVO and IVF conceptuses, C areas, and IC areas. **c** Volcano plot of differentially expressed proteins (DEPs) in conceptuses between the IVO and IVF groups. The red and green dots represent upregulated or downregulated DEPs, respectively (−log_10_(*P*-value) > 1; mean fold change > 2 or < 0.5). IVO-specific proteins were listed in the green box, and the IVF-specific proteins were listed in the red box. **d** Unsupervised hierarchical clustering analysis of the DEPs between the IVO and IVF conceptuses, C areas, and IC areas. **e** Distribution of DEPs with different fold changes in the conceptuses, C areas and IC areas. **f** Diagram of DEPs between the IVO and IVF conceptuses, C areas and IC areas. Representative DEPs common to different samples, and the related functions are presented in the table

The experimental design is presented in Fig. 1 and Additional file 1: Figure S1A. To profile the IVF-associated proteome in conceptuses and their matched endometria at the implantation stage, IVO and IVF blastocysts were transferred to synchronized recipient ewes. On day 17 of pregnancy, which is the end point of the peri-implantation period [50, 51], and is frequently selected to explore the mechanisms of embryo-maternal crosstalk at the implantation stage in ewes [16, 52], filamentous conceptuses and their matched endometrial C areas and IC areas were sampled from each pregnant ewe. In both the IVO and IVF groups, the collected samples were divided into three pools for further proteomic analysis (Additional file 1: Figure S1A). Overall, using LC-ESI-MS/MS, we successfully identified 6374, 7495, 7933, 6162, 7401 and 8456 peptides in IVO-conceptuses, IVO- C areas, IVO-IC areas, IVF-conceptuses, IVF- C areas and IVF-IC areas, respectively. The consistence among biological replicates was evaluated by determining Pearson’s correlation coefficients using the summed peptide intensity values for each protein in the conceptuses, C areas, and IC areas. We found that the Pearson’s correlation coefficient was over 0.8 (Additional file 1: Figure S1B), indicating a general consistency in sample preparation and detection. In addition, as expected, unsupervised hierarchical clustering of the protein intensity profiles also revealed that the endometrial C and IC areas clustered closely together, and the cluster expanded to the conceptuses in IVO or IVF groups (Additional file 1: Figure S1C). In addition, the samples from the IVO and IVF groups clustered separately (Fig. 1b).

Comparative analysis of differentially expressed proteins (DEPs) between the IVF and IVO groups showed that the levels of 207, 295, and 403 proteins were significantly changed in the conceptuses, endometrial C areas, and IC areas, respectively (Additional file 3: Table S2). Notably, we found some proteins were specifically expressed in IVF conceptuses (e.g., CHD8, TSTA3), i.e., they were aberrantly activated in IVF conceptuses; while some proteins were specifically expressed in IVO conceptuses (e.g., NHLRC2, SRI), i.e., they were deficient in IVF conceptuses. (Fig. 1c). Similar results were also observed in the IVF endometrial samples (Additional file 1: Figure S1D). Compared with the IVO conceptuses, 130 DEPs were upregulated and 77 DEPs were downregulated in the IVF conceptuses. In contrast, the comparative analysis of DEPs between the IVO and IVF endometrial samples indicated that a greater proportion of the DEPs were downregulated in the IVF C or IC areas: 133 DEPs were upregulated and 162 DEPs were downregulated in the IVF C areas relative to the IVO C areas; 86 DEPs were upregulated and 137 DEPs were downregulated in the IVF IC areas relative to the IVO IC areas. (Fig. 1d). We further divided the DEPs into subcategories based on their fold changes (FC), and noticed that the proportions of dramatically changed (FC > 4) DEPs in the IVF conceptuses were much higher than those in the IVF endometrial samples, implying that IVF-induced aberrations are more dramatic in embryos and the subsequent changes in the endometria are milder but more diverse (Fig. 1e). Next, the endometrium could be considered as an early sensor of embryo implantation, therefore, we focused on the 72 DEPs common to C and IC areas using Venn diagrams. The dysregulated functions in IVF endometria were related to “cellular response to stress”, “protein metabolism”, “cytoskeleton organization”, “cell proliferation”, “genetic information processing” and “energy metabolism” (Fig. 1f). Furthermore, we found seven common DEPs among the conceptuses, C areas and IC areas, and their functions were involved in “protein metabolism”, “membrane organization”, and “cell redox homeostasis” (Fig. 1f), which indicated that the IVF-ET process might affect these physiological processes in the conceptuses and endometria. Next, functional analysis was performed for the DEPs in the different samples.

### Enhanced energy metabolism, over-proliferation, and depressed methyl metabolism in IVF conceptuses

**Fig. 2:**
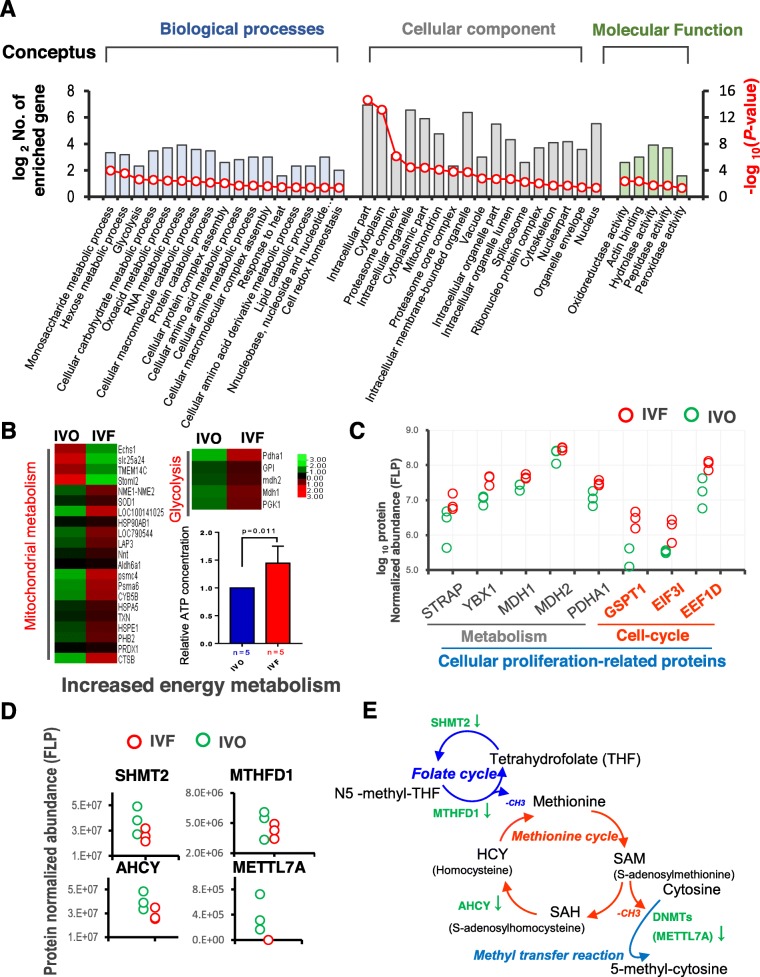
Enhanced energy metabolism, over-proliferation, and depressed methyl metabolism in IVF conceptuses. **a** Classification of GO terms based on functional annotation of ‘biological process’, ‘cellular component’, and ‘molecular function’, using DEPs between IVO and IVF conceptuses. The left ordinate represents the number of DEPs enriched in each term [defined as log_2_ (No. of enriched genes)], and the right ordinate represents the enrichment score [defined as –log_10_(*P*-value)]. **b** Heat map of DEPs associated with mitochondrial metabolism and glycolysis in the IVO and IVF conceptuses. Normalized protein abundance is represented in red (relatively high) and green (relatively low). ATP levels were quantified in the IVO and IVF conceptuses and normalized by the total proteins concentration, “n” represents the biological replicates. **c** Normalized abundance of proteins involved in metabolism and cell cycle in IVO and IVF conceptuses. **d** Normalized abundance of proteins involved in methyl metabolism process. **e** The illustration of dysregulated methyl metabolism process in IVF conceptuses. The downward arrow represents the downregulated DEPs in IVF conceptuses

To gain further insights into the IVF-induced complications in conceptuses by implantation stage, Gene Ontology (GO) annotation using the DEPs between the IVO and IVF conceptuses was performed (Fig. 2a). We found that IVF-induced DEPs were significantly enriched in biological processes related to metabolism of carbohydrates, lipids, and amino acids, especially the GO terms of energy metabolism, such as hexose metabolism, glycolytic process and cellular carbohydrate metabolism. GO terms related to nucleic acid metabolism, such as RNA metabolic process and nucleobase, nucleoside and nucleotide metabolic process, were also enriched, implying the possibility that genetic information processing might be disrupted in the IVF conceptuses. Next, we performed REVIGO analysis to visualize the interactive relationship among the enriched terms. The results showed that metabolism of amino acids, carbohydrates, and nucleic acids were more closely related (Additional file 1: Figure S2A).

Based on these suggestions, we extracted DEPs responsible for mitochondrial functions and glycolysis, which account for the main proportion of energy metabolism of embryos by implantation stage [53–55]. A heat map showed that these DEPs were more abundant in IVF conceptuses. This is in line with the detection of the normalized total ATP concentrations in the IVF and IVO conceptuses, which showed that the total ATP concentration of IVF group was significantly higher than that of the IVO group, indicating that the IVF conceptuses might enhance the energy metabolism. (Fig. 2b). The enhanced energy metabolism led us speculate that IVF conceptuses might undergo increased proliferation, since metabolism is a critical determinant for proliferation during implantation development [56–58]. To test this, we analyzed the DEPs involved in cellular proliferation based on the gene list provided by QuickGO. As expected, IVF conceptuses showed the upregulation in metabolism and cell cycle-related proteins, which might result in enhanced proliferation in IVF conceptuses (Fig. 2c). Phenotypic analysis using the MGI database showed that some of these DEPs are important for early embryonic development (Additional file 1: Figure S2C). These results are partially in agreement with our previous observations of disrupted energy metabolism in IVF embryos [27]. However, this disruption seems to vary among species: In mice, genes involved in mitochondrial energy metabolism were likely to be inhibited in IVF embryos by the implantation stage [13], which was associated with decreased fetal weight throughout gestation [13, 26, 27]. By contrast, proteins associated with energy metabolism and proliferation were upregulated in sheep IVF conceptuses. This might explain the distinct neonatal phenotypes among species following IVF-ET, i.e., intrauterine growth restriction (IUGR) and low birth weight in humans and rodents [59–62], and large offspring syndrome (LOS) in ruminants [63, 64].

Next, using DEPs from the conceptuses as seed nodes, we constructed interaction networks (Additional file 1: Figure S2B). In addition to the enriched terms of macromolecular metabolism, we also identified that the abundance of SUGT1, DNASE2, and TXN and other redox homeostasis-related proteins were upregulated in IVF conceptuses (Additional file 1: Figure S2E), which might be an adaptive change in response to the enhanced metabolism. Our recently published studies have demonstrated that energy metabolism, especially that by mitochondria, is the major source of reactive oxygen species (ROS), and IVF blastocysts are characterized by increased oxidative stress [13, 65]. The present results, using conceptuses at the implantation stage, suggested that redox homeostasis might be consistently dysregulated in IVF embryos during early development.

DNA methylation dynamics is a prominent epigenetic hallmark of early development. Our own work [26], as well as other related studies [8], suggested that the IVF process disrupted the establishment or maintenance of DNA methylation, caused by the inhibited expression of DNA methyltransferases (DNMTs). In the present study, we screened for enzymes involved in DNA methylation modification. Although no detectable changes were observed in the level of DNMTs, we found that the abundance of methyl metabolism-related proteins, such as SHMT2, MTHFD1, AHCY, and METTL7A, which catalyze key steps of methyl metabolism and transfer, decreased in the IVF conceptuses (Fig. 2d), For example, folic acid is the methyl donor of s-adenosylmethionine (SAM), and SAM is the unique active methyl donor of the DNA methylation process. The process is involved in three metabolic cycles: The folate cycle, the methionine cycle, and the methylation/demethylation cycle. In our data, the abundance of MTHFD1 and SHMT2 was reduced in IVF conceptuses, which might decrease the amount of —CH_3_ generated from the folate cycle. This is in accord with our previous observation that the one carbon pool by folate pathway was disrupted in IVF mouse embryos [27]. The expression of AHCY, which plays a role in the methionine cycle, also decreased, which might decrease the amount of —CH_3_ provided by the methionine cycle. Meanwhile, the decreased abundance of METTL7A might lead to a reduced source of 5-methyl-cytosine (Fig. 2e). Among these enzymes, MTHFD1 and AHCY were annotated with phenotypes such as “embryonic lethality prior to/during organogenesis”, “abnormal neural tube closure” and “impaired somite development” (Additional file 1: Figure S2D), implying that the downregulated proteins associated with methyl metabolism might contribute to the impaired development of IVF conceptuses, such as increased early embryonic lethality and impaired fetal neural development, as we reported previously [13]. The present findings, together with previous observations [8, 26], indicated that not only the methyl transfer reactions, but also one-carbon cycle-mediated methyl metabolism, were depressed by IVF processes. These results might explain why embryos or offspring following IVF-ET are associated with global hypo-methylation or loss of imprinting [66–68], and provide a potential strategy to rescue the impaired DNA methylation modifications in IVF embryos by supplementing substrate or precursor of methyl metabolism [69, 70].

### Impaired endometrial remodeling and dysregulated redox homeostasis in IVF endometrial C areas

**Fig. 3:**
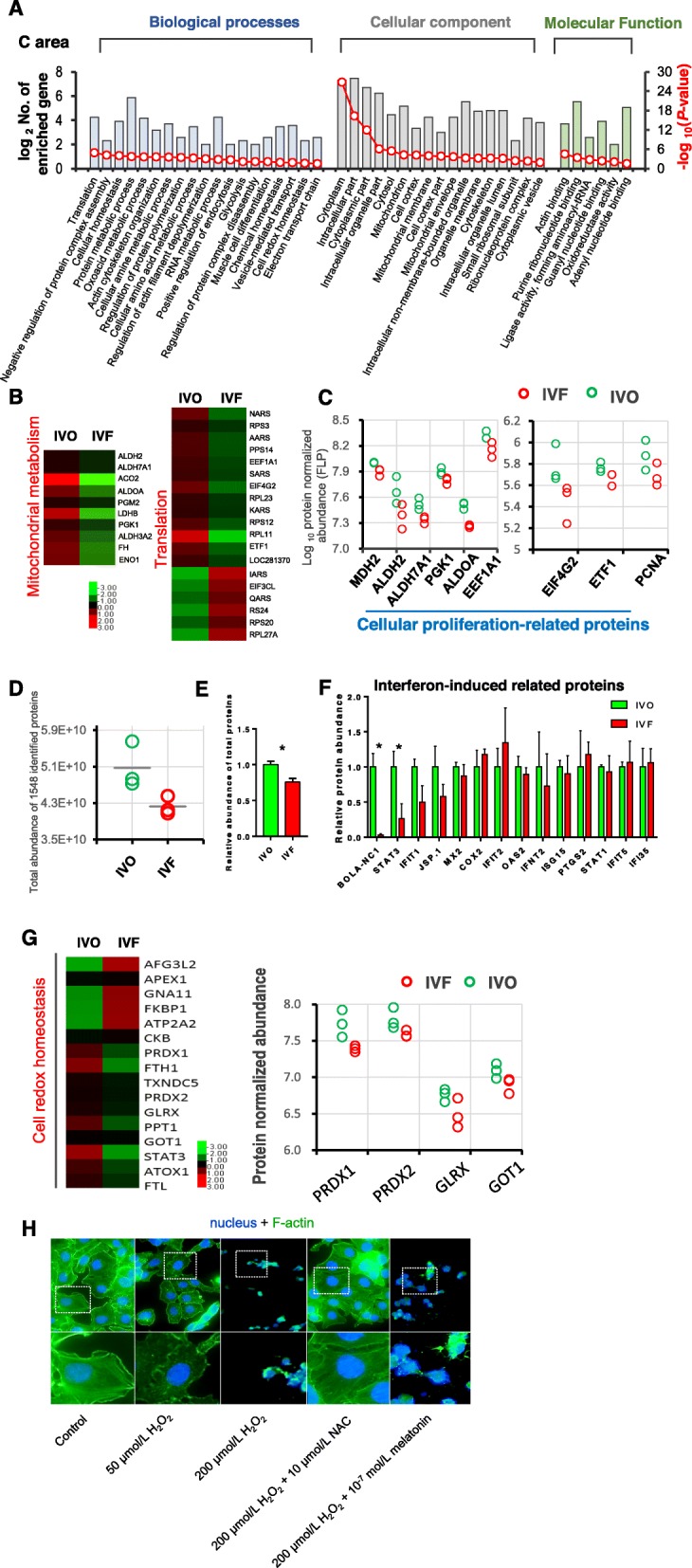
Impaired endometrial remodeling and dysregulated redox homeostasis in IVF endometrial C areas. **a** Classification of GO terms based on functional annotation of ‘biological process’, ‘cellular component’, and ‘molecular function’, using DEPs between IVO and IVF C areas. The left ordinate represents the number of DEPs enriched in each term [defined as log_2_ (No. of enriched genes)], and the right ordinate represents the enrichment score [defined as –log_10_(*P*-value)]. **b** Heat map of DEPs associated with mitochondrial metabolism and translation in the IVO and IVF C areas. Normalized protein abundance is represented in red (relatively high) and green (relatively low). **c** Normalized abundance of proteins involved in cellular proliferation in IVO and IVF C areas. **d** Comparisons of the total abundance of 1548 proteins of the IVO and IVF C area samples. Each circle indicates the total abundance of 1548 proteins of a biological replicate from the IVO or IVF C area samples. **e** Quantitation of total protein concentration per gram of tissue in IVO and IVF C area samples. Data represent the mean ± SEM of three independent biological replicates, *P < 0.05. **f** Normalized abundance of proteins encoded by interferon-induced genes in the IVO and IVF C areas. Data represent the mean ± SEM, * *P* < 0.05. **g** (Right) Heat map of DEPs associated with cell redox homeostasis in the IVO and IVF C areas. Normalized protein abundance is represented in red (relatively high) and green (relatively low). (Left) Normalized abundance of proteins involved in cellular homeostasis in the IVO and IVF C areas. **h** Representative fluorescent images of cell nucleus stained by DAPI (blue) and the cytoskeletal structure stained by phalloidin (green) in human endometrial cancer cells (Ishikawa line) following different treatments

Next, we investigated if changed embryonic development following IVF processes induces an aberrant endometrial response. Functional profiling was performed using DEPs between the IVO and IVF C areas (Fig. 3a). GO annotation showed a similar enrichment pattern to the IVF conceptuses: Energy metabolism, amino acid metabolism, and RNA metabolism-related terms, were significantly represented. These observations were further confirmed by protein-protein interaction network constructions, in which DEPs involved in mitochondrial functions and translation processes were tightly clustered (Additional file 1: Figure S3A). In addition, REVIGO analysis further suggested that GO terms of protein metabolism, cellular amine metabolism, RNA metabolism and translation were more closely related (Additional file 1: Figure S3B). Based on these considerations, we screened DEPs involved in mitochondrial functions and translation processes, because energy metabolism and protein synthesis are primary aspects of endometrial remodeling during pregnancy [34, 71, 72]. Detailed analysis showed that a greater proportion of the DEPs associated with mitochondrial functions and translation were downregulated in IVF endometrial C areas (Fig. 3b). Based on the observation of downregulated translation-related proteins, we first compared the total abundance of the 1548 proteins detected by LC-ESI-MS/MS between the IVO and IVF C areas, and found that the total abundance of the 1548 identified proteins was decreased in each replicate of the IVF endometrial C samples (Fig. 3d). Next, we measured the total protein contents of the collected samples, the results of which showed that significantly lower amounts of total protein were detected in the IVF endometrial C areas, compared with that in their IVO counterparts (Fig. 3e). In addition, we observed the lower abundance of inducing proliferation related-proteins in IVF C areas than in IVO C areas, including MDH2, ALDH2, ALDH7A1, PGK1, ALDOA, EEF1A1, EIF4G2, ETF1, and PCNA (Fig. 3c). This implied that the proliferation of IVF endometrial C areas might be inhibited. Endometrial proliferation, the hallmark of the remodeling response to conceptus-derived signals and maternal hormone signals, is essential to establish endometrial receptivity by the implantation stage in different species [34, 73]. The important role of DEPs associated with these terms in impaired endometrial receptivity could be revealed by MGI annotation: ALDH2 and EPRS were annotated with “abnormal embryo size”; EPRS, EIF4G2, ARAS, and KARS were annotated with “embryonic lethality prior to/during organogenesis” (Additional file 1: Figure S3C).

The proposed hypothesis of impaired endometrial response was also supported by the expression patterns of proteins encoded by interferon-induced genes. In the IVF endometrial C areas, a proportion of these proteins were not upregulated (Fig. 3f). In ruminant species, interferon τ (IFNτ), secreted by the trophectoderm, is the primary signal for pregnancy recognition [74, 75]. IFNτ acts on the endometria to reduce the expression of the estrogen receptor and oxytocin receptor, thus suppressing the uterine luteolytic mechanism, and maintaining progesterone secretion to provide a receptive environment for conceptuses development [16]. In the present study, the protein abundance of BOLA-NC1 (non-classical MHC class I antigen) was reduced by 29 fold. In humans, HLAG (non-classical MHC-1 molecule) plays an important role in embryo implantation and acts as critical tolerogenic mediator for embryo-maternal crosstalk [76]. In cattle, BOLA-NC1 might play a role in early embryo survival and embryo-maternal immune tolerance through interacting with natural killer (NK) cells [77, 78]. The protein abundance of STAT3 (signal transducer and activator of transcription 3) was also downregulated. In mouse models with mutant STAT3 [79], or chemically inhibited STAT3 signaling [80], endometrial receptivity was significantly impaired, leading to implantation failure or embryonic lethality immediately after implantation. Other proteins encoded by interferon-induced genes that are essential for successful implantation in ewes [22], such as IFIT1, JSP-1, and MX2, also showed the lower abundance in the IVF C areas.

Among the enriched terms and pathways identified using functional profiling, we also noted cellular homeostasis and cytoskeleton. Cellular homeostasis is very importance to maintain a relative stable intracellular environment, including PH, media composition, and oxygen. Our data suggested that the levels of certain key enzymes of the glutathione/glutathione peroxidase (GSH/GPX) system were reduced in the IVF C areas (Fig. 3g). Previous studies in mice, ruminants, and humans reported an essential role of GSH/GPX in maintaining endometrial redox homeostasis by protecting against oxidative stress [81]. The inhibited enzymes of the GSH/GPX system, together with the aberrant expression of cytoskeleton-related proteins in the IVF C areas, led us to ask if these complications are functionally associated. To test this, we detected the cytoskeleton organization of *in vitro* cultured human endometrial cancer cells (Ishikawa line) under the chemical-induced oxidative stress. A previous report showed that H_2_O_2_ impaired cytoskeleton organization in a dose-dependent manner [82]. Similarly, our data indicated that 50 μmol/L H_2_O_2_ exposure significantly disrupted the F-actin organization, revealed by decreased fluorescence intensity of FITC-phalloidin staining. In addition, 200 μmol/L H_2_O_2_ exposure lead to a severely degenerated F-actin organization, and necrotic morphology. By supplementation with NAC at 10 μmol/L, the rate-limiting precursor for GSH synthesis, the severe F-actin damage induced by 200 μmol/L H_2_O_2_ exposure was rescued. By contrast, the rescue effect was not observed after supplementation with melatonin at 10^− 7^ mol/L, the physiological dosage for scavenging ROS [14] (Fig. 3h). These results suggested that GSH might play a unique role in protecting endometrial redox homeostasis that cannot be substituted by other antioxidants, thus the impaired GSH/GPX system might contribute largely to the aberrant remodeling of the IVF endometrial C areas.

### Disrupted protein homeostasis and impaired ECM interaction in IVF endometrial IC areas

**Fig. 4:**
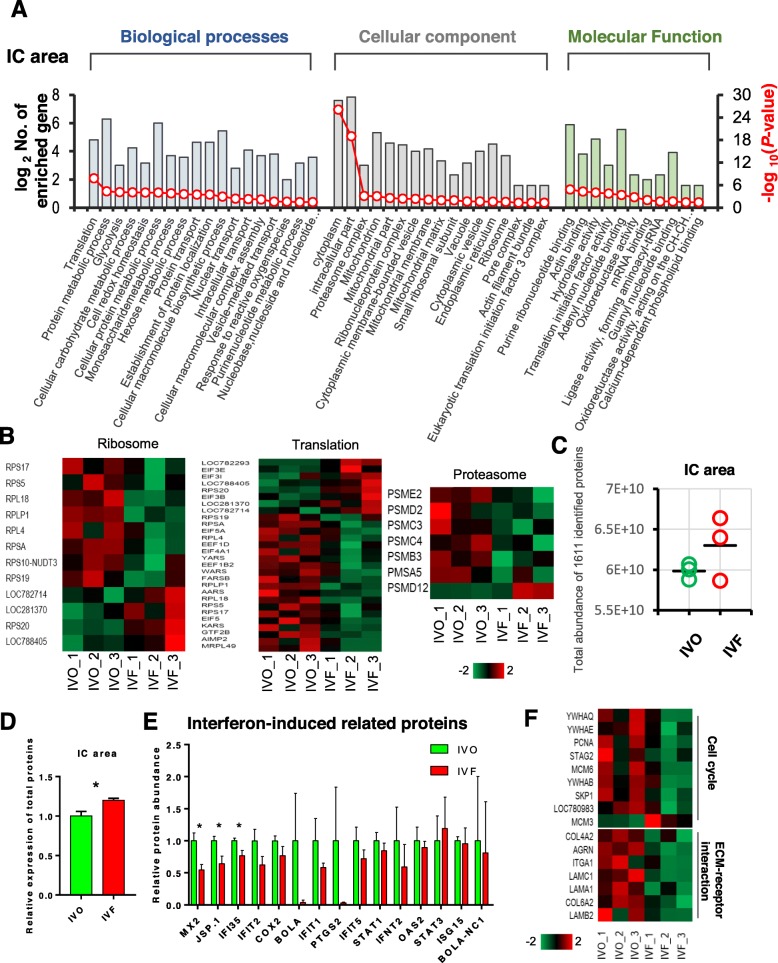
Disrupted protein homeostasis and impaired ECM interaction in IVF endometrial IC areas. **a** Classification of GO terms based on functional annotation of ‘biological process’, ‘cellular component’, and ‘molecular function’, using DEPs between the IVO and IVF IC areas. The left ordinate represents the number of DEPs enriched in each term [defined as log_2_ (No. of enriched genes)], and the right ordinate represents the enrichment score [defined as –log_10_(*P*-value)]. **b** Heat map of DEPs associated with ribosome, translation, and proteasome in the IVO and IVF IC areas. Z-score normalized protein abundance is represented in red (relatively high) and green (relatively low). **c** Comparisons of the total abundance of 1611 proteins of the IVO and IVF C area samples. Each circle indicates the total abundance of 1611 proteins in a biological replicate from the IVO or IVF IC area samples. **d** Quantitation of total protein concentration per gram of tissue in IVO and IVF C area samples. Data represent the mean ± SEM of three independent biology replicates, *P < 0.05. **e** Normalized abundance of proteins encoded by interferon-induced genes in the IVO and IVF IC area samples. Data represent the mean ± SEM, * *P* < 0.05. **f** Heat map of DEPs associated with the cell cycle and ECM receptor in the IVO and IVF IC areas. Z-score normalized protein abundance is represented in red (relatively high) and green (relatively low)

Successful implantation in ruminants depends on synergistic functions of the endometrium C and IC areas. The C areas of the endometrium are the sites of superficial attachment and placentation in ewes, while the IC areas contain large numbers of branched and coiled uterine glands that synthesize, secrete and transport a variety of molecules essential to the development of the conceptuses [22, 25]. Thus, we next compared the proteomic profiles between the IVO and IVF endometrial IC areas. As expected, we found a series of IVF-associated changes in the IC areas that differed from those observed in the C areas. Based on the functional annotations by GO, REVIGO, and STRING network construction, a cascade of terms involved in protein synthesis/degradation were enriched, such as “translation”, “protein metabolic process”, “cellular macromolecule synthetic process”, “small ribosome subunit”, and “proteasome complex”. (Fig. 4a, Additional file 1: Figure S4A, and S4B). Then we screened the expression patterns of proteins associated with these terms. The heat map indicated that many DEPs functionally associated with translation and degeneration of proteins were dysregulated. Notably, a greater proportion of DEPs associated with proteasome systems were downregulated in the IVF IC areas (Fig. 4b), suggesting disrupted protein homeostasis in IVF endometrial IC areas. This observation is in line with the result of the comparison of the total abundance of 1611 proteins detected by LC-ESI-MS/MS between the IVO and IVF C areas (Fig. 4c), as well as the BCA protein assay of total expressed proteins (Fig. 4d).

Furthermore, an impaired response to IFNτ was observed in the IVF IC areas, similar to that observed in the IVF C areas. Many proteins encoded by interferon-induced genes were not upregulated, or showed a decreasing tendency in the IVF IC areas (Fig. 4e). In sheep, MX2 expression rapidly increased in response to IFNτ-inducing by implantation, and was thought to regulate the immune system [19, 83]. In addition, JSP.1 and IFI35 are involved in immune response. JSP.1 is related to presentation of foreign antigens to the immune system. The lower abundance of MX2, JSP.1, and IFI35 in the IVF C areas might lead to dysregulated endometrial immune remodeling, which is essential to prepare future maternal immune tolerance [84], establish endometrial receptivity and the growth of the conceptus by the implantation stage [85, 86].

In addition, the impaired response of IC areas to the signals from the conceptuses was also in line with the downregulated proteins related to extracellular matrix (ECM) organization, proliferation, and energy metabolism (Fig. 4f, Additional file 1: Figure S4C). It has been well-documented that in ruminants, endometrial glands in the IC areas undergo extensive hyperplasia and hypertrophy during early pregnancy, presumably to meet the increasing demands of the developing conceptus for uterine histotroph [87, 88]. Proliferation and ECM interaction are functionally associated and support endometria remodeling [89]. In addition, the important role of ECM proteins in successful implantation were also reported in mice [90] and humans [91], as revealed by influencing a series of cellular behaviors that are essential for implantation, e.g., cell migration, cell growth, cell survival, cell proliferation, angiogenesis, and invasion [92]. Our previous works also indicated that the lower abundance of ECM proteins is associated with pregnancy loss caused by the poor endometrial receptive state [22].

## Conclusion

We profiled the proteome of the IVF conceptuses and their matched endometria, aiming at understanding the mechanism of IVF-induced aberrant embryo-maternal crosstalk during early pregnancy. By functionally profiling the IVF conceptuses, we found that DEPs related to energy metabolism and proliferation were upregulated in IVF conceptuses, which might indicate the enhanced proliferation in the IVF conceptuses. This might be explained by the compensation effect that occurs in IVF embryos. Indeed, the metabolic compensation was thought to be causatively associated with LOS in IVF ruminant offspring [93]. Interestingly, the proteins related to one-carbon cycle-mediated methyl metabolism, which provide methyl groups for methyl transfer reactions of DNA methylation, were downregulated in IVF embryos, which might indicate impaired methyl metabolism. Considering the important role of DNA methylation modification in supporting embryonic or fetal development, impaired methyl metabolism might compromise IVF embryos.

By contrast, the functional profiling of IVF-matched endometria showed the aberrant expression of proteins related to energy metabolism, proliferation, cytoskeleton organization, protein hemostasis, EMC interaction, and the antioxidant system, all of which are essential to establish endometrial receptivity. More importantly, the IVF-matched endometria displayed the reduced abundance of proteins encoded by interferon-induced genes, which might result in a reduced response to pregnancy recognition signals in both the C and IC areas.

**Fig. 5:**
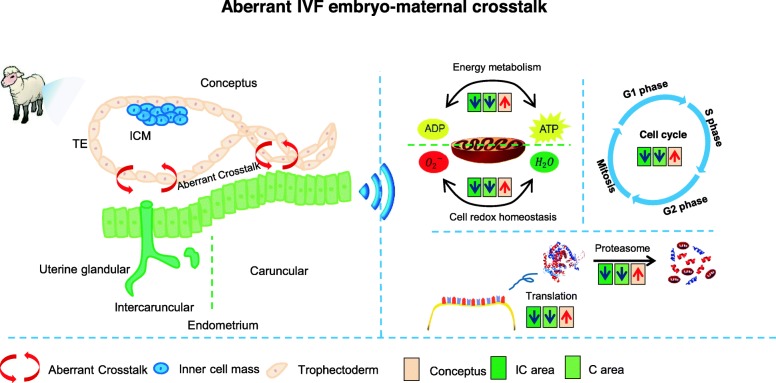
The illustration of cellular and molecular processes of aberrant IVF embryo-maternal crosstalk in ewes. The upward red arrows represent processes that were enriched with upregulated DEPs. The downward blue arrows represent processes that were enriched with downregulated DEPs. In ruminants, IVF-matched endometrium (both C and IC areas) showed disruptions in energy metabolism, cell cycle, protein homeostasis, and cell redox homeostasis, all of which are essential to establish endometrial receptivity. The changed endometrial receptivity is functionally associated with a poor response to IVF conceptuses; in turn, the impaired IVF conceptuses will undergo metabolic and proliferative compensation

Based on these findings, we proposed a model for the disruption of embryo-maternal crosstalk in IVF-ET (Fig. 5). In ruminants, IVF-ET embryos would undergo metabolic and proliferative compensation by upregulating related protein expression by the implantation stage, which might be causatively associated with the LOS phenotype from mid-gestation to the perinatal stage. However, the abnormal state of IVF embryos might further lead to an impaired endometrial response to pregnancy recognition signals, which is essential for successful implantation. Depressed endometrial receptivity would further impair fetal development following implantation. This concept suggests that in addition to enhancing IVF embryo quality by optimizing culture systems, improving the maternal uterine environment will also benefit the pregnancy outcome following IVF-ET, thereby providing a practical strategy in both animal reproductive management and clinical ART.

## Supplementary information

**Additional file 1: Figure S1. (a)** Strategy for samples pooling. Conceptuses are presented as an example. In the IVO (or IVF) group, the samples of 36 (or 18) conceptuses were equally and randomly divided into three pools as independent biological replicates, each pooled sample was detected twice as technical replicates. **(b)** Pearson’s Correlation coefficient between the randomly selected two biological replicates using the summed peptide intensity values for each protein in the conceptus, C area and IC area samples to confirm the reliability of the processes of sample collection, LC-ESI-MS/MS detection and proteomic analysis. **(c)** Unsupervised clustering of the protein expression patterns of each biological replicate of the conceptus, C area and IC area samples from the IVO and IVF groups. **(d)** Volcano plot of DEPs in the C areas, IC areas and conceptuses between the IVO and IVF groups. The red and green dots represent upregulated or downregulated DEPs, respectively (−Log_10_(*P*-value) > 1; mean fold change > 2 or < 0.5). **Figure S2. (a)** Graph visualization of biological processes (BPs) enriched in the IVF conceptuses based on REVIGO analysis. The bigger circle represents the more clustered genes. The gradation of color represents the significance level. Functionally associated BPs are linked. **(b)** Interaction networks of DEPs between the IVO and IVF conceptuses were created by a web-based search of the STRING database. Boxed regions represent tightly interconnected functional clusters. **(c-d)** The representative MGI phenotypes associated with embryonic development annotated with DEPs related to metabolism and cell cycle (c) and methyl metabolism (d). **(e)** Heat map of DEPs related to redox homeostasis in the IVO and IVF conceptuses. Normalized protein abundance is represented in red (relatively high) and green (relatively low). The table shows the representative MGI phenotypes of DEPs in redox homeostasis. **Figure S3. (a)** Interaction networks of DEPs between IVO and IVF conceptuses were created by a web-based search of the STRING database. Boxed regions represent tightly interconnected functional clusters **(b)**. Graphical visualization of biological processes (BPs) enriched in IVF C areas based on REVIGO analysis. The bigger circle represents the more clustered genes. The gradation of the color represents the significance level. Functionally associated BPs are linked. **(c)** The representative MGI phenotypes associated with embryonic development annotated with DEPs related to metabolism and translation. **Figure S4. (a)** Graphical visualization of biological processes (BPs) enriched in IVF IC areas based on REVIGO analysis The bigger circle represents the more clustered genes. The gradation of the color represents the significance level. Functionally associated BPs are linked. **(b)** Interaction networks of DEPs between the IVO and IVF IC areas are created by a web-based search of the STRING database. Boxed regions represent tightly interconnected functional clusters. **(c)** Heat map of DEPs involved in Glycolysis/Gluconeogenesis in the IC areas. The level of Log_2_(IVF/IVO) is represented in red (relatively high) and green (relatively low).

**Additional file 2: Table S1.** The protein quantification values for all datasets produced.

**Additional file 3: Table S2.** Differentially expressed proteins (DEPs) between the IVF and IVO in conceptuses, endometrial C areas, and IC areas.

## Data Availability

All data generated or analyzed during this study are included in this published article [and its supplementary information files].

## Abbreviations

ACN: Acetonitrile
AGC: Automatic gain control
AI: Artificial insemination
ART: Assisted reproductive technology
BSA: Bovine serum albumin
C areas: Caruncular areas
CID: Collision induced dissociation
DEPs: Differentially expressed proteins
DNMTs: DNA methyltransferases
DTT: Dithiothreitol
ECM: Extracellular matrix
EDTA: Ethylenediaminetetraacetic acid
EGF: Epidermal growth factor
ESI: Electrospray ionization
FA: Formic acid
FBS: Fetal bovine serum
FC: Fold change
FDR: False discovery rate
FITC: Fluorescein isothiocyanate labeled
FSH: Follicle stimulating hormone
GO: Gene Ontology
GSH/GPX: Glutathione/glutathione peroxidase
HPLC: High performance liquid chromatography
IAM: Iodoacetamide
IC areas: Intercaruncular areas
IFN-τ: Interferon τ
IUGR: Intrauterine growth restriction
IVF-ET: *In vitro* fertilization and embryo transfer
IVM: *In vitro* maturation
IVO: *In vivo*
LC: Liquid chromatography
LC-ESI-MS/MS: Liquid chromatography-electrospray ionization-tandem mass spectroscopy
LH: Luteinizing hormone
LOS: Large offspring syndrome
MS: Mass spectrometry
NAC: N-acetyl-L-cysteine
P/S: Penicillin and streptomycin
PBS: Phosphate-buffered saline
PMSF: Phenylmethanesulfonyl fluoride
ROS: Reactive oxygen species
SAM: S-adenosylmethionine
SOF: Synthetic oviductal fluid
TCA: Trichloroacetic acid
XICs: Extracted ion currents

## References

[1] Mansouri-Attia N, Sandra O, Aubert J, Degrelle S, Everts RE, Giraud-Delville C (2009). Endometrium as an early sensor of in vitro embryo manipulation technologies. Proc Natl Acad Sci.

[2] Achache H, Revel A (2006). Endometrial receptivity markers, the journey to successful embryo implantation. Hum Reprod Update.

[3] Diedrich K, Fauser BCJM, Devroey P, Griesinger G (2007). Evian annual reproduction (EVAR) workshop group. The role of the endometrium and embryo in human implantation. Hum Reprod Update.

[4] Bauersachs S, Ulbrich SE, Zakhartchenko V, Minten M, Reichenbach M, Reichenbach H-D (2009). The endometrium responds differently to cloned versus fertilized embryos. Proc Natl Acad Sci U S A.

[5] Emiliani S, Delbaere A, Devreker F, Englert Y (2005). Embryo-maternal interactive factors regulating the implantation process: implications in assisted reproductive. Reprod BioMed Online.

[6] Holm P, Walker SK, Seamark RF (1996). Embryo viability, duration of gestation and birth weight in sheep after transfer of in vitro matured and in vitro fertilized zygotes cultured in vitro or in vivo. Reproduction.

[7] Kruip TAM, den Daas JHG (1997). In vitro produced and cloned embryos: effects on pregnancy, parturition and offspring. Theriogenology.

[8] Ptak GE, D’Agostino A, Toschi P, Fidanza A, Zacchini F, Czernik M (2013). Post-implantation mortality of in vitro produced embryos is associated with DNA methyltransferase 1 dysfunction in sheep placenta. Hum Reprod.

[9] Tan K, An L, Miao K, Ren L, Hou Z, Tao L (2016). Impaired imprinted X chromosome inactivation is responsible for the skewed sex ratio following in vitro fertilization. Proc Natl Acad Sci.

[10] Driver AM, Peñagaricano F, Huang W, Ahmad KR, Hackbart KS, Wiltbank MC (2012). RNA-Seq analysis uncovers transcriptomic variations between morphologically similar in vivo- and in vitro-derived bovine blastocysts. BMC Genomics.

[11] Schieve LA, Meikle SF, Ferre C, Peterson HB, Jeng G, Wilcox LS (2002). Low and very low birth weight in infants conceived with use of assisted reproductive technology. N Engl J Med.

[12] Tan K, An L, Wang SM, Wang XD, Zhang ZN, Miao K (2015). Actin disorganization plays a vital role in impaired embryonic development of in vitro-produced mouse preimplantation embryos. PLoS One.

[13] Ren L, Wang Z, An L, Zhang Z, Tan K, Miao K (2015). Dynamic comparisons of high-resolution expression profiles highlighting mitochondria-related genes between in vivo and in vitro fertilized early mouse embryos. Hum Reprod.

[14] Tian X, Zhang L, Tan D, Wang F, Liu G, Reiter RJ (2013). Melatonin promotes the in vitro development of pronuclear embryos and increases the efficiency of blastocyst implantation in murine. J Pineal Res.

[15] Ali AA, Bilodeau JF, Sirard MA (2003). Antioxidant requirements for bovine oocytes varies during in vitro maturation, fertilization and development. Theriogenology.

[16] Bazer FW, Spencer TE, Ott TL (1997). Interferon tau: A novel pregnancy recognition signal. Am J Reprod Immunol.

[17] Macklon NS, Brosens JJ (2014). The human endometrium as a sensor of embryo Quality1. Biol Reprod.

[18] Lundeberg T, Parasassi T, Pittaluga E, Brunelli R (2017). N-acetyl-L-cysteine for use in in vitro fertilization.

[19] Gray CA, Abbey CA, Beremand PD, Choi Y, Farmer JL, Adelson DL (2006). Identification of endometrial genes regulated by early pregnancy, progesterone, and interferon tau in the ovine Uterus1. Biol Reprod.

[20] Koch JM, Ramadoss J, Magness RR (2010). Proteomic profile of uterine luminal fluid from early pregnant ewes. J Proteome Res.

[21] Wang Y, Wang C, Hou Z, Miao K, Zhao H, Wang R (2013). Comparative analysis of proteomic profiles between endometrial caruncular and intercaruncular areas in ewes during the peri-implantation period. J Anim Sci Biotechnol.

[22] Zhao H, Sui L, Miao K, An L, Wang D, Hou Z (2015). Comparative analysis between endometrial proteomes of pregnant and non-pregnant ewes during the peri-implantation period. J Anim Sci Biotechnol.

[23] Mamo S, Mehta JP, Forde N, McGettigan P, Lonergan P (2012). Conceptus-endometrium crosstalk during maternal recognition of pregnancy in cattle. Biol Reprod.

[24] Berendt FJ, Fröhlich T, Schmidt SEM, Reichenbach H-D, Wolf E, Arnold GJ (2005). Holistic differential analysis of embryo-induced alterations in the proteome of bovine endometrium in the preattachment period. Proteomics.

[25] Mansouri-Attia N, Aubert J, Reinaud P, Giraud-Delville C, Taghouti G, Galio L (2009). Gene expression profiles of bovine caruncular and intercaruncular endometrium at implantation. Physiol Genomics.

[26] Sui L, An L, Tan K, Wang Z, Wang S, Miao K (2014). Dynamic proteomic profiles of in vivo- and in vitro-produced mouse Postimplantation Extraembryonic tissues and Placentas1. Biol Reprod.

[27] Nie J, An L, Miao K, Hou Z, Yu Y, Tan K (2013). Comparative analysis of dynamic proteomic profiles between in vivo and in vitro produced mouse embryos during postimplantation period. J Proteome Res.

[28] Chae J-I, Kim J, Lee SG, Jeon Y-J, Kim D-W, Soh Y (2011). Proteomic analysis of pregnancy-related proteins from pig uterus endometrium during pregnancy. Proteome Sci.

[29] Rashid NA, Lalitkumar S, Lalitkumar PG, Gemzell-Danielsson K (2011). Endometrial receptivity and human embryo implantation. Am J Reprod Immunol.

[30] Salamonsen LA, Edgell T, Rombauts LJF, Stephens AN, Robertson DM, Rainczuk A (2013). Proteomics of the human endometrium and uterine fluid: a pathway to biomarker discovery. Fertil Steril.

[31] Lee KY, DeMayo FJ (2004). Animal models of implantation. Reproduction.

[32] Barry JS, Anthony RV (2008). The pregnant sheep as a model for human pregnancy. Theriogenology.

[33] Spencer TE, Johnson GA, Bazer FW, Burghardt RC (2004). Implantation mechanisms: insights from the sheep. Reproduction.

[34] Igwebuike UM (2009). A review of uterine structural modifications that influence conceptus implantation and development in sheep and goats. Anim Reprod Sci.

[35] Schwanhäusser B, Busse D, Li N, Dittmar G, Schuchhardt J, Wolf J (2013). Erratum: corrigendum: global quantification of mammalian gene expression control. Nature.

[36] De Matos DG, Gasparrini B, Pasqualini SR, Thompson JG (2002). Effect of glutathione synthesis stimulation during in vitro maturation of ovine oocytes on embryo development and intracellular peroxide content. Theriogenology.

[37] De Matos DG, Furnus CC, Moses DF, Martinez AG, Matkovic M (1996). Stimulation of glutathione synthesis of in vitro matured bovine oocytes and its effect on embryo development and freezability. Mol Reprod Dev.

[38] Graumann J, Hubner NC, Kim JB, Ko K, Moser M, Kumar C (2008). Stable isotope labeling by amino acids in cell culture (SILAC) and proteome quantitation of mouse embryonic stem cells to a depth of 5,111 proteins. Mol Cell Proteomics.

[39] Cox J, Mann M (2008). MaxQuant enables high peptide identification rates, individualized p.p.b.-range mass accuracies and proteome-wide protein quantification. Nat Biotechnol.

[40] Li GZ, JPC V, Silva JC, Golick D, Gorenstein MV, Geromanos SJ (2009). Database searching and accounting of multiplexed precursor and product ion spectra from the data independent analysis of simple and complex peptide mixtures. Proteomics.

[41] Feng J, Naiman DQ, Cooper B (2007). Probability-based pattern recognition and statistical framework for randomization: modeling tandem mass spectrum/peptide sequence false match frequencies. Bioinformatics.

[42] Waanders LF, Chwalek K, Monetti M, Kumar C, Lammert E, Mann M (2009). Quantitative proteomic analysis of single pancreatic islets. Proc Natl Acad Sci U S A.

[43] Kim Y, Jeon J, Mejia S, Yao CQ, Ignatchenko V, Nyalwidhe JO (2016). Targeted proteomics identifies liquid-biopsy signatures for extracapsular prostate cancer. Nat Commun.

[44] Lapek JD, Greninger P, Morris R, Amzallag A, Pruteanu-Malinici I, Benes CH (2017). Detection of dysregulated protein-association networks by high-throughput proteomics predicts cancer vulnerabilities. Nat Biotechnol.

[45] Huang DW, Sherman BT, Tan Q, Kir J, Liu D, Bryant D (2007). DAVID Bioinformatics Resources: Expanded annotation database and novel algorithms to better extract biology from large gene lists. Nucleic Acids Res.

[46] Huang DW, Sherman BT, Lempicki RA (2009). Systematic and integrative analysis of large gene lists using DAVID bioinformatics resources. Nat Protoc.

[47] Huntley RP, Sawford T, Mutowo-Meullenet P, Shypitsyna A, Bonilla C, Martin MJ (2015). The GOA database: Gene Ontology annotation updates for 2015. Nucleic Acids Res.

[48] Franceschini A, Szklarczyk D, Frankild S, Kuhn M, Simonovic M, Roth A (2013). STRING v9.1: Protein-protein interaction networks, with increased coverage and integration. Nucleic Acids Res.

[49] Supek F, Bošnjak M, Škunca N, Šmuc T (2011). REVIGO summarizes and visualizes long lists of gene ontology terms. PLoS One.

[50] Wan P-C, Bao Z-J, Wu Y, Yang L, Hao Z-D, Yang Y-L (2011). αvβ3 integrin may participate in Conceptus attachment by regulating morphologic changes in the endometrium during Peri-implantation in ovine. Reprod Domest Anim.

[51] Spencer T, Burghardt R, Johnson G, Bazer F (2004). Conceptus signals for establishment and maintenance of pregnancy. Anim Reprod Sci.

[52] Song G, Satterfield MC, Kim J, Bazer FW, Spencer TE (2008). Gastrin-releasing peptide (GRP) in the ovine uterus: regulation by interferon tau and Progesterone1. Biol Reprod.

[53] Zhou W, Choi M, Margineantu D, Margaretha L, Hesson J, Cavanaugh C (2012). HIF1α induced switch from bivalent to exclusively glycolytic metabolism during ESC-to-EpiSC/hESC transition. EMBO J.

[54] Leese H (1995). Metabolic control during preimplantation mammalian development. Hum Reprod Update.

[55] Harvey AJ (2007). The role of oxygen in ruminant preimplantation embryo development and metabolism. Anim Reprod Sci.

[56] Krisher RL, Prather RS (2012). A role for the Warburg effect in preimplantation embryo development: metabolic modification to support rapid cell proliferation. Mol Reprod Dev.

[57] Folmes CDL, Terzic A (2015). Metabolic determinants of embryonic development and stem cell fate. Reprod Fertil Dev.

[58] Ramalho-Santos J, Varum S, Amaral S, Mota PC, Sousa AP, Amaral A (2009). Mitochondrial functionality in reproduction: from gonads and gametes to embryos and embryonic stem cells. Hum Reprod Update.

[59] Moise J, Laor A, Armon Y, Gur I, Gale R (1998). The outcome of twin pregnancies after IVF. Hum Reprod.

[60] Pinborg A, Loft A, Schmidt L, Langhoff-Roos J, Andersen AN (2004). Maternal risks and perinatal outcome in a Danish national cohort of 1005 twin pregnancies: the role of in vitro fertilization. Acta Obstet Gynecol Scand.

[61] Nassar AH, Usta IM, Rechdan JB, Harb TS, Adra AM, Abu-Musa AA (2003). Pregnancy outcome in spontaneous twins versus twins who were conceived through in vitro fertilization. Am J Obstet Gynecol.

[62] Cai L, Izumi S, Koido S, Uchida N, Suzuki T, Matsubayashi H (2006). Abnormal placental cord insertion may induce intrauterine growth restriction in IVF-twin pregnancies. Hum Reprod.

[63] Chen Z, Robbins KM, Wells KD, Rivera RM (2013). Large offspring syndrome. Epigenetics.

[64] Chen Zhiyuan, Hagen Darren E., Elsik Christine G., Ji Tieming, Morris Collin James, Moon Laura Emily, Rivera Rocío Melissa (2015). Characterization of global loss of imprinting in fetal overgrowth syndrome induced by assisted reproduction. Proceedings of the National Academy of Sciences.

[65] REN L, ZHANG C, TAO L, HAO J, TAN K, MIAO K (2017). High-resolution profiles of gene expression and DNA methylation highlight mitochondrial modifications during early embryonic development. J Reprod Dev.

[66] Mann MRW (2004). Selective loss of imprinting in the placenta following preimplantation development in culture. Development.

[67] Wright KP, Brown L, Casson PR, Johnson JV, Brown S (2008). ART conditions are associated with global genomic hypomethylation at the blastocyst stage in mice. Fertil Steril.

[68] Katari S, Turan N, Bibikova M, Erinle O, Chalian R, Foster M (2009). DNA methylation and gene expression differences in children conceived in vitro or in vivo. Hum Mol Genet.

[69] Vollset SE, Gjessing HK, Tandberg A, Rønning T, Irgens LM, Baste V (2005). Folate Supplementation and Twin Pregnancies. Epidemiology.

[70] Laanpere M, Altmäe S, Stavreus-Evers A, Nilsson TK, Yngve A, Salumets A (2010). Folate-mediated one-carbon metabolism and its effect on female fertility and pregnancy viability. Nutr Rev.

[71] Chen Q, Zhang A, Yu F, Gao J, Liu Y, Yu C (2015). Label-free proteomics uncovers energy metabolism and focal adhesion regulations responsive for endometrium receptivity. J Proteome Res.

[72] Włodarek J, Jaśkowski J, Nowak W, MIKUŁA JAN, Olechnowicz J (2011). Development of ovarian follicles, quality of oocytes and fertility of cows in view of a negative energy balance in the transition period. Med Weter.

[73] Paulson RJ (2011). Hormonal induction of endometrial receptivity. Fertil Steril.

[74] Ashworth CJ, Bazer FW (1989). Changes in ovine Conceptus and endometrial function following Asynchronus embryo transfer or administration of Progesterone1. Biol Reprod.

[75] Farin CE, Imakawa K, Roberts RM (1989). In situ localization of mRNA for the interferon, ovine Trophoblast Protein-1, during early embryonic development of the sheep. Mol Endocrinol.

[76] Le Bouteiller P (2002). Commentary major breakthrough in the HLA-G debate: occurrence of pregnancy in human depends on the HLA-G status of preimplantation embryos. Eur J Immunol.

[77] O’Gorman GM (2010). Naib a Al, Ellis SA, Mamo S, O’Doherty AM, Lonergan P, et al. regulation of a bovine nonclassical major histocompatibility complex class I gene Promoter1. Biol Reprod.

[78] Araibi EH, Marchetti B, Dornan ES, Ashrafi GH, Dobromylskyj M, Ellis SA (2006). The E5 oncoprotein of BPV-4 does not interfere with the biosynthetic pathway of non-classical MHC class I. Virology.

[79] Takeda K, Noguchi K, Shi W, Tanaka T, Matsumoto M, Yoshida N (1997). Targeted disruption of the mouse Stat3 gene leads to early embryonic lethality. Proc Natl Acad Sci U S A.

[80] Catalano RD, Johnson MH, Campbell EA, Charnock-Jones DS, Smith SK, Sharkey AM (2005). Inhibition of Stat3 activation in the endometrium prevents implantation: a nonsteroidal approach to contraception. Proc Natl Acad Sci.

[81] Serviddio G, Loverro G, Vicino M, Prigigallo F, Grattagliano I, Altomare E (2002). Modulation of endometrial redox balance during the menstrual cycle: relation with sex hormones. J Clin Endocrinol Metab.

[82] Zhao Y, Davis HW (1998). Hydrogen peroxide-induced cytoskeletal rearrangement in cultured pulmonary endothelial cells. J Cell Physiol.

[83] Ott TL, Yin J, Wiley AA, Kim H-T, Gerami-Naini B, Spencer TE (1998). Effects of the estrous cycle and early pregnancy on uterine expression of mx protein in sheep (Ovis aries)1. Biol Reprod.

[84] Lédée N, Prat-Ellenberg L, Chevrier L, Balet R, Simon C, Lenoble C (2017). Uterine immune profiling for increasing live birth rate: a one-to-one matched cohort study. J Reprod Immunol.

[85] Hannan NJ, Evans J, Salamonsen LA (2011). Alternate roles for immune regulators: establishing endometrial receptivity for implantation. Expert Rev Clin Immunol.

[86] Ott TL, Kamat MM, Vasudevan S, Townson DH, Pate JL. Maternal immune responses to conceptus signals during early pregnancy in ruminants. Anim Reprod. 2014;11(n3):237–45.

[87] Stewart M. David, Johnson Greg A., Gray C. Allison, Burghardt Robert C., Schuler Linda A., Joyce Margaret M., Bazer Fuller W., Spencer Thomas E. (2000). Prolactin Receptor and Uterine Milk Protein Expression in the Ovine Endometrium During the Estrous Cycle and Pregnancy1. Biology of Reproduction.

[88] Bazer FW (1975). Uterine protein secretions: relationship to development of the conceptus. J Anim Sci.

[89] Gray CA, Bartol FF, Tarleton BJ, Wiley AA, Johnson GA, Bazer FW (2001). Developmental biology of uterine Glands1. Biol Reprod.

[90] Woessner JF (1991). Matrix metalloproteinases and their inhibitors in connective tissue remodeling. FASEB J.

[91] Fisher SJ, Damsky CH (1993). Human cytotrophoblast invasion. Semin Cell Biol.

[92] Reddy KVR, Mangale SS (2003). Integrin receptors: the dynamic modulators of endometrial function. Tissue Cell.

[93] Niemann H, Korsawe K, Lemme E, Lucas-Hahn A, Herrmann D, Wrenzycki C (2004). Gene expression patterns in in vitro-produced and somatic nuclear transfer-derived preimplantation bovine embryos: relationship to the large offspring syndrome?. Anim Reprod Sci.

