# Supplementary material for: The proteome of IVF-induced aberrant embryo-maternal crosstalk by implantation stage in ewes
Source: J Anim Sci Biotechnol. 2020 Jan 14;11:7. doi: 10.1186/s40104-019-0405-y (PMC6958772; doi:10.1186/s40104-019-0405-y)
Supplement: Supplementary file 1 — Additional file 1: Figure S1. (a) Strategy for samples pooling. Conceptuses are presented as an example. In the IVO (or IVF) group, the samples of 36 (or 18) conceptuses were equally and randomly divided into three pools as independent biological replicates, each pooled sample was detected twice as technical replicates. (b) Pearson’s Correlation coefficient between the randomly selected two biological replicates using the summed peptide intensity values for each protein in the conceptus, C area and IC area samples to confirm the reliability of the processes of sample collection, LC-ESI-MS/MS detection and proteomic analysis. (c) Unsupervised clustering of the protein expression patterns of each biological replicate of the conceptus, C area and IC area samples from the IVO and IVF groups. (d) Volcano plot of DEPs in the C areas, IC areas and conceptuses between the IVO and IVF groups. The red and green dots represent upregulated or downregulated DEPs, respectively (−Log10(P-value) > 1; mean fold change > 2 or < 0.5). Figure S2. (a) Graph visualization of biological processes (BPs) enriched in the IVF conceptuses based on REVIGO analysis. The bigger circle represents the more clustered genes. The gradation of color represents the significance level. Functionally associated BPs are linked. (b) Interaction networks of DEPs between the IVO and IVF conceptuses were created by a web-based search of the STRING database. Boxed regions represent tightly interconnected functional clusters. (c-d) The representative MGI phenotypes associated with embryonic development annotated with DEPs related to metabolism and cell cycle (c) and methyl metabolism (d). (e) Heat map of DEPs related to redox homeostasis in the IVO and IVF conceptuses. Normalized protein abundance is represented in red (relatively high) and green (relatively low). The table shows the representative MGI phenotypes of DEPs in redox homeostasis. Figure S3. (a) Interaction networks of DEPs between IVO and IVF concept [file 40104_2019_405_MOESM1_ESM.pdf]

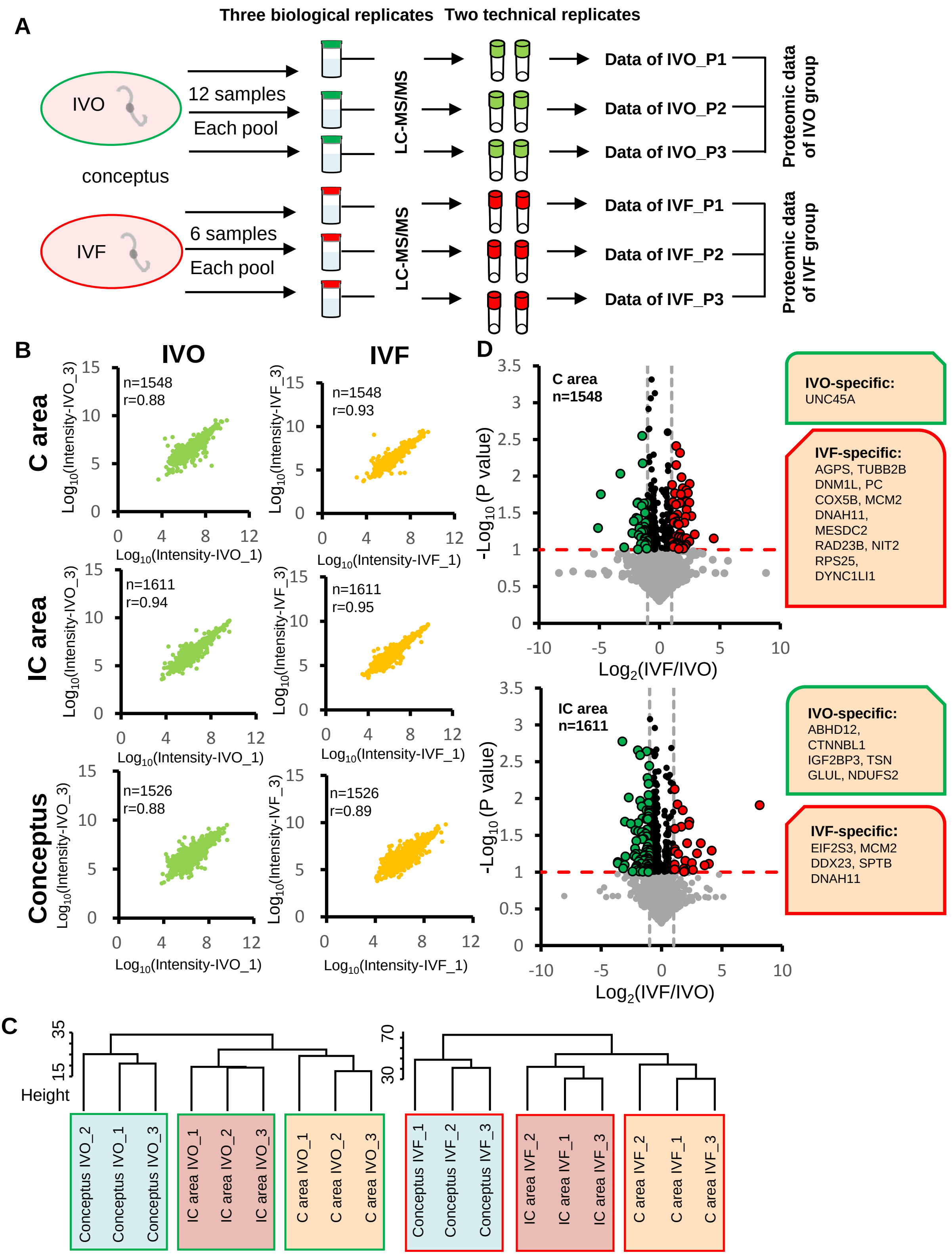
A
Three biological replicates
Two technical replicates
IVO
conceptus
IVF
12 samples
Each pool
6 samples
Each pool
LC-MS/MS
Data of IVO_P1
Data of IVO_P2
Data of IVO_P3
Data of IVF_P1
Data of IVF_P2
Data of IVF_P3
Proteomic data of IVO group
Proteomic data of IVF group
B
IVO
IVF
C area
IC area
Conceptus
n=1548
r=0.88
r=0.93
n=1611
r=0.94
r=0.95
n=1526
r=0.88
r=0.89
Log10(Intensity-IVO_3)
Log10(Intensity-IVO_1)
Log10(Intensity-IVF_3)
Log10(Intensity-IVF_1)
D
C area
n=1548
IC area
n=1611
-Log10(P value)
Log2(IVF/IVO)
IVO-specific:
UNC45A
IVF-specific:
AGPS, TUBB2B
DNM1L, PC
COX5B, MCM2
DNAH11,
MESDC2
RAD23B, NIT2
RPS25,
DYNC1LI1
IVO-specific:
ABHD12,
CTNNBL1
IGF2BP3, TSN
GLUL, NDUFS2
IVF-specific:
EIF2S3, MCM2
DDX23, SPTB
DNAH11
C
Height
Conceptus IVO_2
Conceptus IVO_1
Conceptus IVO_3
IC area IVO_1
IC area IVO_2
IC area IVO_3
C area IVO_1
C area IVO_2
C area IVO_3
Conceptus IVF_1
Conceptus IVF_2
Conceptus IVF_3
IC area IVF_2
IC area IVF_1
IC area IVF_3
C area IVF_2
C area IVF_1
C area IVF_3

A

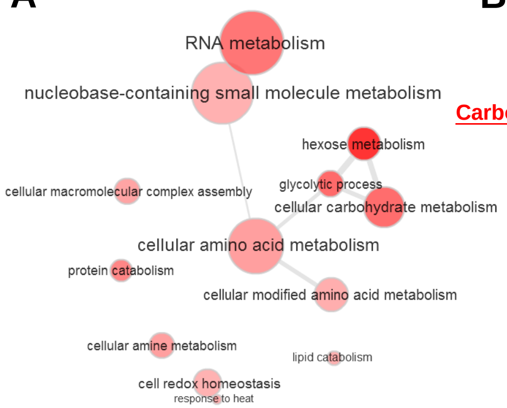

B

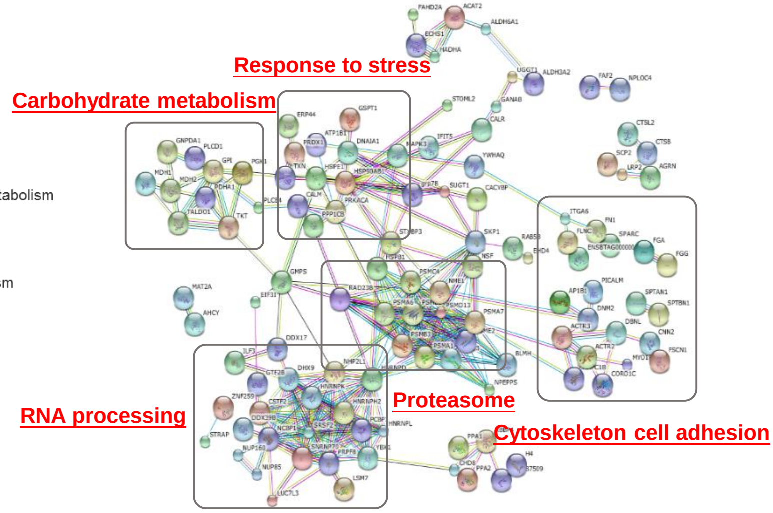

C

Representative phenotypes associated with embryonic development

Symbol	Name	Phenotypes
PDHA1	pyruvate dehydrogenase E1 alpha 1	Decreased embryo size; Embryonic growth retardation; Embryonic lethality
PGM3	phosphoglucomutase 3	Embryonic lethality between implantation and somite formation
GNPDA1	glucosamine-6-phosphate deaminase 1	Abnormal embryo size; Embryonic lethality prior to organogenesis; Embryonic lethality prior to tooth bud stage.
GPI	glucose phosphate isomerase 1	Abnormal embryonic tissue morphology; Embryonic lethality between implantation and placentation; Embryonic lethality
STRAP	serine/threonine kinase receptor associated protein	Abnormal embryo turning Embryonic lethality during organogenesis
SUGT1	SGT1, suppressor of G2 allele of SKP1	Embryonic lethality prior to organogenesis

D

Symbol	Name	Phenotypes
MTHFD1	Methylenetetrahydrofolate dehydrogenase	Absent somites; Impaired somite development; Decreased embryo size; Embryonic growth retardation; Embryonic lethality during organogenesis; Abnormal neural tube closure
AHCY	S-adenosylhomocysteine hydrolase	Embryonic lethality prior to organogenesis; Preweaning lethality

E

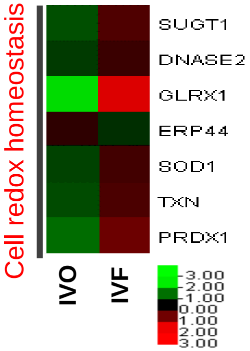

Symbol	Name	Phenotypes
SUGT1	SGT1, suppressor of G2 allele of SKP1	Embryonic lethality prior to organogenesis
DNASE2	deoxyribonuclease II alpha	Abnormal apoptosis; Lethality during fetal growth
TXN	thioredoxin 1	Abnormal trophoblast layer morphology; Embryonic growth arrest; Embryonic lethality between implantation and somite formation, Absent inner cell mass proliferation; Failure of blastocyst to hatch from the zona pellucida

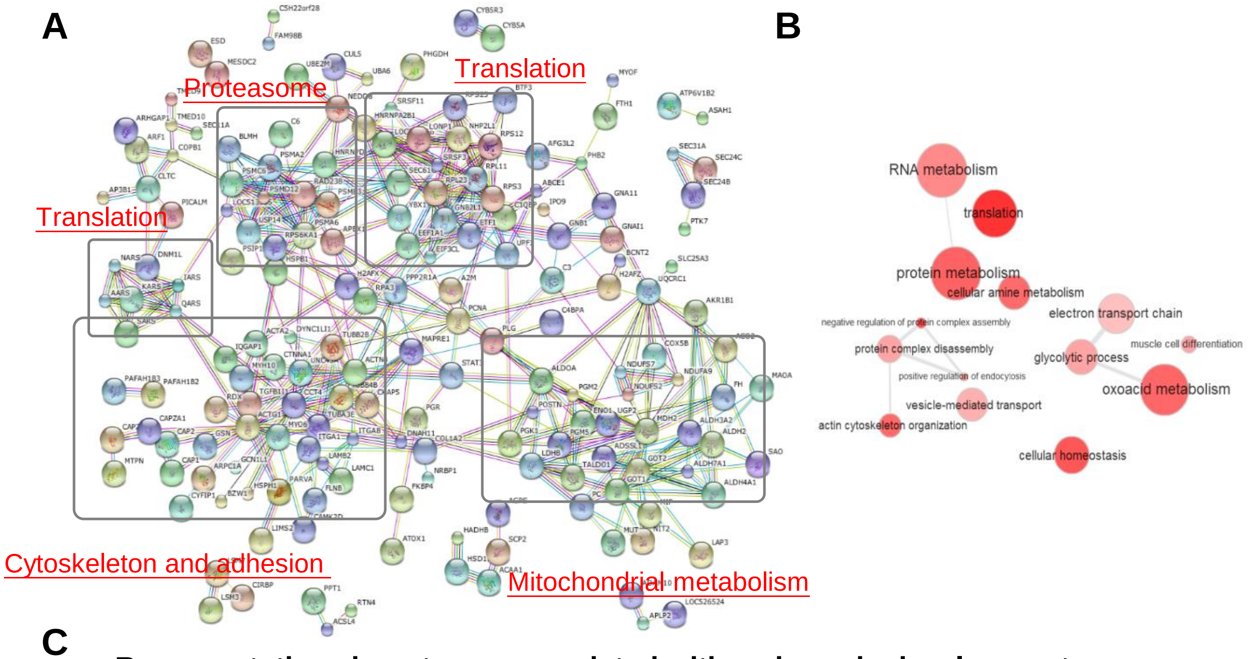

C

Representative phenotypes associated with embryonic development

Symbol	Name	Phenotypes
Aldh2	Aldehyde dehydrogenase 2, mitochondrial	Decreased litter size
Eprs	Glutamyl-prolyl-tRNA synthetase	Abnormal embryo size;<br>Embryonic lethality prior to / during organogenesis;
Eif4g2	Eukaryotic translation initiation factor 4, gamma 2	Embryonic lethality prior to / during organogenesis;<br>Embryonic growth retardation
Aars	alanyl-tRNA synthetase	Embryonic lethality prior to / during organogenesis
Rpl27a	lysyl-tRNA synthetase	Embryonic lethality
Fh1	Fumarate hydratase 1	Embryonic lethality between implantation and somite formation
Kars	lysyl-tRNA synthetase	Embryonic lethality prior to / during organogenesis

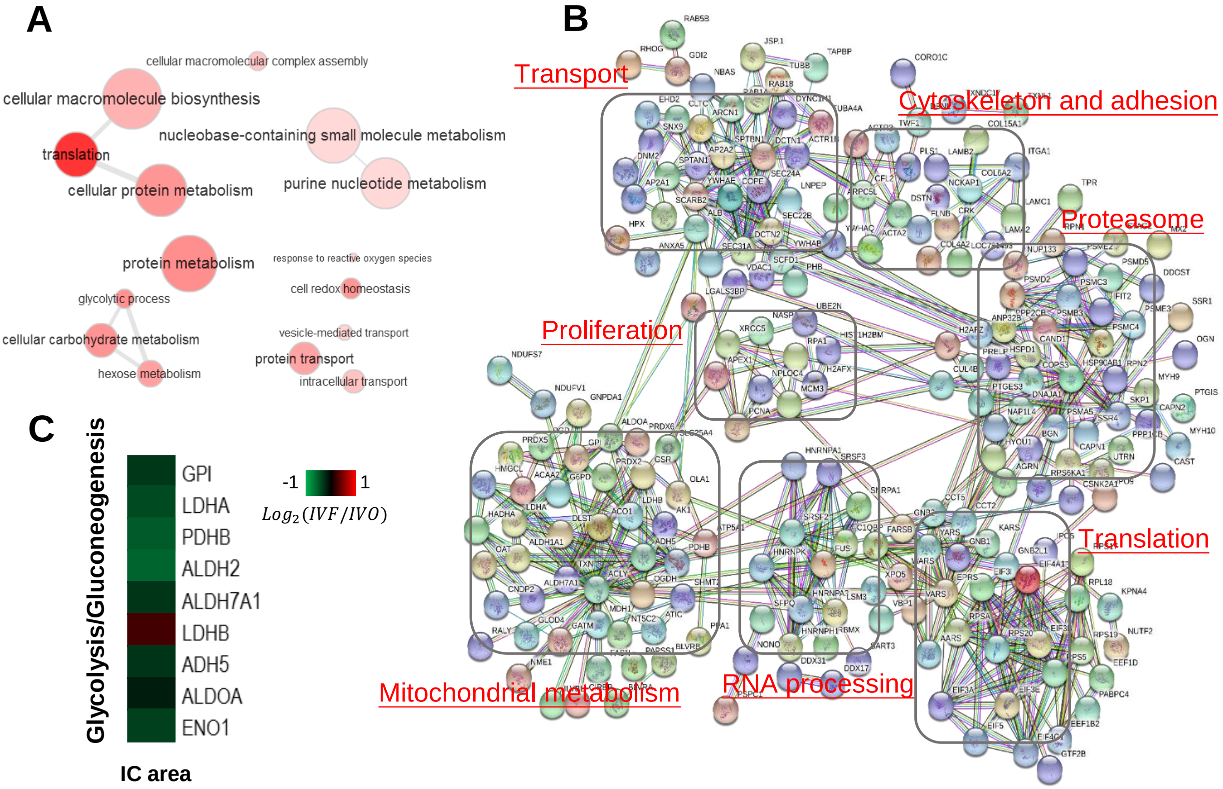

A
cellular macromolecular complex assembly
cellular macromolecule biosynthesis
translation
nucleobase-containing small molecule metabolism
cellular protein metabolism
purine nucleotide metabolism
protein metabolism
response to reactive oxygen species
cell redox homeostasis
glycolytic process
cellular carbohydrate metabolism
hexose metabolism
vesicle-mediated transport
protein transport
intracellular transport
B
Transport
Cytoskeleton and adhesion
Proteasome
Proliferation
Translation
Mitochondrial metabolism
RNA processing
C
Glycolysis/Gluconeogenesis
GPI
LDHA
PDHB
ALDH2
ALDH7A1
LDHB
ADH5
ALDOA
ENO1
IC area
-1
1
$Log_2(IVF/IVO)$
